# Editorial: Women in cancer metabolism: 2021/2022

**DOI:** 10.3389/fonc.2022.1042349

**Published:** 2022-11-30

**Authors:** Tuuli Kaambre

**Affiliations:** Laboratory of Chemical Biology, National Institute of Chemical Physics and Biophysics, Tallinn, Estonia

**Keywords:** cancer, metabolism, metastasis, mechanisms, metabolites, cancer models *in vivo*

Women have participated in research and made important contributions to it since the beginning of the history of science. On the other hand, women’s participation has often been hindered by the prevalent patriarchal worldview; higher positions are mostly held by men, while women are predominantly in teaching positions. In recent years, the role of women in science has begun to increase, including in cancer research. This collection of articles supports women scientists to make their work more visible in the scientific community.

Cancer cells need energy for survival, proliferation, and the formation of metastases. Several mechanisms are involved in these processes. Today’s research is focused on how essential nutrients such as glucose, lactate, fatty acids, and glutamine are utilized by the cells, cellular metabolomics, and also on the regulation and communication of metabolic pathways. The need to find new tumor drug targets and biomarkers has become very important and these studies allow getting closer to that goal.

## Breast and ovarian cancer studies – from woman to woman

In this issue, three female scientists have contributed to the study of tumors that directly attack women like breast and ovarian cancer.

The article by Ruggiero et. al., where they studied the lifetime of the intracellular water as the tumor biomarker, is dedicated to breast cancer described relaxometric measurements using FFC-NMR on tumor specimens *in vivo* using 4T1 and doxorubicin-resistant 4T1R cell lines and by using a model of Balb/c Mice Bearing 4T1 tumors *in vitro*. These experiments resulted in a decrease in kio (cellular water efflux rate constant) during the doxorubicin treatment which decreased the activity of Na+/K+-ATPase (validated in the control experiments on the cellular uptake of Rb ions). The results reported show that kio can be considered a non-invasive, early, and predictive biomarker for the identification of responsive patients immediately after the first doxorubicin treatment.

Penkert with colleagues described systemic metabolic alterations that exist in BRCA1+ individuals independent of cancer incidence. This group detected one single metabolite, pyruvate, and two metabolite ratios involving pyruvate, lactate, and a metabolite of yet unknown structure (RI1984, MS studies showing this a sugar acid), significantly altered between the cohorts of BRCA1+ women and 72 age-matched female controls. The results allow us to assume that constitutional alterations in energy metabolism may be involved in the etiology of BRCA1-associated breast cancer.

Ovarian cancer is one of the most aggressive malignancies worldwide and He et al. studied the aberrant cholesterol metabolism of this tumor. The authors give a systematic overview of the key proteins involved in cholesterol and oxysterol metabolism in ovarian cancer, including the rate-limiting enzymes in cholesterol biosynthesis, and the proteins involved in cholesterol uptake, storage, and trafficking. Both cholesterol and oxysterol reprogram the cellular microenvironment, high levels of these sterols may be immunosuppressive.

## Energy metabolism of glioblastoma

Glioblastoma (GMB) is still a tumor with a very poor prognosis and complicated treatment; this tumor is also rather radioresistant and therefore understanding the metabolism type of this tumor is essential. One very important metabolite for tumor cells is lactate; its effect on glioblastoma has been described by McKelvey et al. They showed, that glucose and FAO metabolic pathways are similar among two glioblastoma cell culture subtypes, and enzymes of the glycolytic and FAO pathways are upregulated in GBM tumors compared to normal brain tissues. These cellular energetic pathways are closed down by the inhibitor of glycolysis - dichloroacetate, and the partial fatty acid oxidation inhibitor ranolazine (Rano). Based on the work, it became clear that dual targeting of glycolytic and FAO metabolic pathways (or/and adjuvant to standard chemoradiation) provides new avenues in treatment principles of cancer.

The importance of lactate in tumors is also evident in the following work Longhitano et al. described the role of monocarboxylate transporter-1 and hydroxycarboxylic acid receptor 1 role of tumor growth and progression in glioblastoma *in vitro* and *in vivo* models of zebrafish. They showed that lactate increased MCT1 and HCAR1 expression; lactate modulated epithelial-mesenchymal transition protein markers E-Cadherin and β-Catenin and therefore proliferation, migration, and colony formation ability.

Harland and her colleagues described the metabolic models of characteristics of glioma stem-like cells. They showed three possible models: 1) the cancer stem cell hierarchical model; 2) rthe stochastic/clonal evolution model and 3) the plasticity model which looks to be a more veracious description of the development and behavior of these cells. This article is a very comprehensive review of almost all metabolic aspects of glioblastoma interaction between metabolism and GSCs which may create an opportunity for future strategies for overcoming GSC resistance.

## Metabolic aspects of the lung cancer

Another example of tumors with a very poor prognosis is lung cancer. Daks et al. showed, that the knockdown of Set7/9 methyltransferase (lysine-specific methyltransferase) in human NSCLC cell models upregulates several important glycolytic enzymes like hexokinase, aldolase, and lactate dehydrogenase together with their key activators—oncogenes c-Myc and HIF1A - at both transcriptional and protein levels. Both activators are well-known coordinators of glycolysis, oxygen consumption, one-carbon metabolism, and the metabolism of glutamine and lipids upon tumorigenesis. Set7/9 methylates the p53 protein, which leads to p53 stabilization *via* inactivation of SIRT1 by its methylation.

Another article by Evers et al. sheds light on metabolic changes in lung cancer describes the epithelial-mesenchymal transition in relationship with extracellular ATP (eATP) and TGF-beta. eATP induced several EMT-related changes in metabolic pathways including the rearrangement of the cytoskeletal architecture, glycolysis, glutaminolysis, ROS, and individual metabolic changes similar to those induced by TGF-β. The extracellular ATP-induced gene expression is not identical to the TGF-β-initiated expression, but they are similar. The same phenomenon is shown also in downregulated genes, primarily epithelial genes. As the cancer cells can use either TGF-β or eATP or both, this may be the reason for metabolic flexibility. This paper is a new and interesting direction to open novel targets to inhibit EMT in cancer.

## Advances in other tumors

Colorectal cancer (CRC) is one of the three most common cancers worldwide; it is the fourth reason for cancer deaths on average and the most frequent cause for non-smokers. The ideal screening biomarkers would be detectable by a non-invasive technique that allows rapid determination of specific metabolites in blood or urine. Mika et al. described rearrangements of blood and tissue fatty acid profiles in this cancer. Their study confirms significant changes in the fatty acid profiles in serum and tumor tissue of CRC patients, the clearest change in patients with CRC is the presence of very long chain fatty acids, which increased 5-10 times. The study about fatty acid profiles is providing a new diagnostic approach for use in the clinic.

One serious challenge for oncologists is the high-grade metastatic chondrosarcomas which are very often not amenable to surgery and are resistant to chemotherapy and radiation therapy. Micaily et al. with her colleagues described profoundly metabolic pathways and targets in chondrosarcoma like the relationship between signaling *via* isocitrate dehydrogenase 1 and 2, hedgehog, PI3K-mTOR-AKT, and SRC, as well as histone acetylation and angiogenesis. As a result of their research, they summarized potential metabolic and therapeutic targets in chondrosarcoma.

Pita-Grisanti et al. described the potential and risk of bacterial siderophores in cancer. Siderophores are iron-chelating small molecules (MW ~ 500–1500 Daltons) essential to mammalian iron homeostasis. Siderophores is a previously almost unexplored field of cancer studies. Cancer cells need increased iron concentration for proliferation. During exogenous siderophore treatment, siderophores bind iron and decrease the levels of free iron available for bacteria. These molecules can interact with the immune system, are linked to pathogen virulence, are signaling molecules, can bind other metals, could be iron chelating anticancer agents, and mediators of drug delivery. However, this topic needs a lot of further research.

We can only hope that the rapid progress in tumor research will continue and that interesting times with fascinating results are ahead.

## Author contributions

The author confirms being the sole contributor of this work and has approved it for publication.

## Conflict of interest

The author declares that the research was conducted in the absence of any commercial or financial relationships that could be construed as a potential conflict of interest.

## Publisher’s note

All claims expressed in this article are solely those of the authors and do not necessarily represent those of their affiliated organizations, or those of the publisher, the editors and the reviewers. Any product that may be evaluated in this article, or claim that may be made by its manufacturer, is not guaranteed or endorsed by the publisher.

